# Hospital Expenditure: The Commissariat

**Published:** 1896-03-21

**Authors:** 


### The Institutional Workshop.

### HOSPITAL EXPENDITURE.—THE COMMISSARIAT.

### XII.—THE COST OF WHAT PATIENTS PROVIDE.

It is usual, in comparing the cost of provisions at different hospitals, to call attention to the fact that many which provide every article for the patient spend no more per bed on provisions than institutions in which tea, sugar, and butter are not supplied. Hence, it almost seems necessary to infer that by some unfathomable process of hospital finance hundreds of persons can be supplied with these necessaries two or three times a day free of cost. The apparent mystery is, however, simple enough. The cost of tea, sugar, and butter is a large item, and can never be anything else. Yet, however large, it is but one among many far larger strands which go to make up the total, and its true bearing on the average cost per bed can be detected in no other way than by eliminating all extraneous expenses and narrowing the issue, as in the previous article we attempted to do, to the actual cost of the food consumed by the patient alone.

In reckoning the actual cost of this time-honoured metropolitan tax on patients (it is rare almost to extinction out of London), it is necessary to remember that in all hospitals, from a certain proportion of patients, it is never exacted at all. It must not for a moment be supposed that in the event of a patient being too poor to buy tea and butter he is left to drink cold water and eat dry bread. Such a state of things would, of course, be intolerable to all concerned. In practice the hospital pays if the patient is unable to do so, or in case the hospital rule is not to be violated, a compromise is effected through the medium of the Samaritan Fund. The proportion of patients to whom this aid is extended varies according to the locality of the hospital and the season of the year from 5 to 20 per cent. Taking all the year round, it may be estimated that at least 10 per cent. of the patients in hospitals where this demand is made are found to be unable to meet it.

There is no difficulty in estimating the cost of the patients' tea, sugar, and butter. As regards tea, the usual allowance is a pint twice a day, and one ounce of tea will make six pints. Each patient then will consume 2⅓ oz. of tea per week. The price paid for the patients' tea ranges from 10d. to 2s. per lb. Taking it at 1s. 4d., which is about the average, and for which very decent tea can be obtained in bulk, the weekly cost is about 2½d. a head. There remains ½ lb. of sugar at 2d. per lb., and ½ lb. of butter at about 1s. 2d. per lb. The total expense is from 10½d. to 1s. per week, and may be considered fairly to represent an extra £2 10s. on each occupied bed. If hospital accounts were systematically analysed in the statement prepared for public consideration, it would not be difficult for the authorities of institutions in which patients are not supplied with tea, sugar, and butter, to show that a corresponding saving is effected. At present the most curious anomalies are presented without explanation.

A large institution like the Leeds General Hospital, for instance, with an average of over 355 occupied beds, will expend on the patients' tea, sugar, and butter between £800 and £900 a year, and yet succeed in showing a yearly average per bed for provisions less by £10 or £12 than University or the London Hospital, where only one-tenth of the patients are provided with these necessaries, and that from a separate fund. It is hazardous to be offering even a conjecture in such a matter, but it may be pointed out that the difference of 3s. a week in the board of each nurse, and of from 10s. to 15s. in the board of each resident medical officer, such as a comparison between prices at metropolitan and provincial institutions has shown to exist, would far more than suffice to wipe out the surplus spent on the patients' tea and butter. A few more nurses, a few extra servants, an additional officer or two would have the same effect; but how far more satisfactory for all concerned if this were clearly defined, if the cost of each individual were clearly set forth, and the public were granted an opportunity of making up the sum needed to purchase the additional commodities.

Whether or no it be desirable to make a fixed charge for all patients who can reasonably afford it is a matter which hospital authorities will have sooner or later to decide. But pending this decision the rule which compels the poor while inmates of the institution partly to board themselves is a mere relic of long-discredited makeshifts, and entails on all hospitals where it is in force a serious loss of dignity. The appropriation of a part of the Samaritan Fund for purchasing these necessaries is another makeshift of an even less excusable nature. These funds are never large, and are nearly always inadequate to perform the work for which they are subscribed, viz., the purchase of tools, warm clothing, &c., and the payment of journeys for outgoing patients in a weak and often destitute condition. It is in every way unsuitable that the cost of the necessary articles of food for the patient while under treatment should be deducted from moneys bestowed for the purpose of easing the burden of life for him when he leaves the hospital. And if it be pleaded that the money is given partly with the aim of supplying these articles of food, consider whether a more ill-sounding expedient could be devised than to tack on to the appeals already made on behalf of the hospital an extra one avowedly made for the purpose of covering deficiencies in the patient's bill of fare.

On every ground of common sense and of economy of labour each inmate of the institution should receive alike every necessary article of food during the time he is under its roof.

